# Exchanging and managing *in*-*vitro* elite germplasm to combat Cassava Brown Streak Disease (CBSD) and Cassava Mosaic Disease (CMD) in Eastern and Southern Africa

**DOI:** 10.1007/s12571-018-0779-2

**Published:** 2018-03-17

**Authors:** Silver Tumwegamire, Edward Kanju, James Legg, Rudolph Shirima, Salehe Kombo, Geoffrey Mkamilo, Kiddo Mtunda, Karoline Sichalwe, Heneriko Kulembeka, Innocent Ndyetabura, Haji Saleh, Robert Kawuki, Titus Alicai, Gerald Adiga, Ibrahim Benesi, Albert Mhone, Anabela Zacarias, Sofrimento Fenias Matsimbe, Theresia Munga, Elijah Ateka, Lynet Navangi, Midatharahally Narasegowda Maruthi, Francis Mwatuni, George Ngundo, Maureen Mwangangi, Edward Mbugua, Joseph Ndunguru, Cyprian Rajabu, Deogratius Mark

**Affiliations:** 1 International Institute of Tropical Agriculture (IITA), PO Box 34441, Dar es Salaam, Tanzania; 2 Department of Agricultural Research and Development, PO Box 2066, Dar es Salaam, Tanzania; 3 Zanzibar Agricultural Research Institute, PO Box 159, Zanzibar, Tanzania; 4 National Agricultural Research Organization, PO Box 295, Entebbe, Uganda; 5 Department for Agricultural Research Services, Chitedze Research Station, PO Box 158, Lilongwe, Malawi; 6 Instituto de Investigacao Agraria de Mocambique, PO Box 3658, Maputo, Mozambique; 7 Kenya Agricultural and Livestock Research Organization, PO Box 57811 – 00200, Nairobi, Kenya; 8 Jomo Kenyatta University of Agriculture and Technology, PO Box 62,000 – 00200, Nairobi, Kenya; 9 Natural Resources Institute, University of Greenwich, Chatham Maritime, Kent, UK; 10 Kenya Plant Inspectorate Services, Plant Quarantine and Biosecurity Station, Muguga, PO Box 49592 – 00100, Nairobi, Kenya; 11 Genetic Technologies International Limited, PO Box 47430 – 00100, Nairobi, Kenya; 12 Mikocheni Agricultural Research Institute, PO Box 6226, Dar es Salaam, Tanzania

**Keywords:** Exchange, In-vitro, Germplasm, CBSD and CMD

## Abstract

Cassava varieties resistant to cassava mosaic disease (CMD) and cassava brown streak disease (CBSD) are needed for the food and income security of the rural poor in eastern and southern Africa (ESA). The International Institute of Tropical Agriculture led five national cassava breeding programs (Malawi, Mozambique, Kenya, Tanzania and Uganda) in virus-cleaning and exchanging elite cassava germplasm resistant to both diseases. This paper documents the experiences and lessons learned from the process. Thirty-one clones (25 elite, two standard and four national) were submitted by the five breeding programs to the Natural Resources Institute and Kenya Plant Health Inspectorate Services for virus cleaning and indexing. Subsequently, ca 75 invitro virus-indexed plantlets per clone were sent to Genetic Technologies International Limited (GTIL), a private tissue culture (TC) lab in Kenya, and micro-propagated to produce ≥1500 plantlets. After fulfilling all the formal procedures of germplasm exchange between countries ≥300 plantlets per clone were sent to each partner country. National check clones susceptible to CMD/CBSD were sent only to their countries of origin. In each country, the in-vitro plantlets were acclimatized under screen house conditions and transferred to clean isolated sites for field multiplication. All the clones were cleaned of the viruses, except Tomo. The cleaning process was slow for F19-NL, NASE1, and Kibandameno and TC micro-propagation at GTIL was less efficient for Pwani, Tajirika, NASE1, and Okhumelela than for the other clones. Difficulties in cleaning recalcitrant clones affected the timeline for establishing the multi-site evaluation trials in target countries. The initiative is the one of the kind to successfully clean and exchange elite germplasm as a joint action to combat CBSD in ESA. Adequate preparation in terms of infrastructure and personnel are critical to successfully receiving and adapting the indexed in-vitro plants as new germplasm.


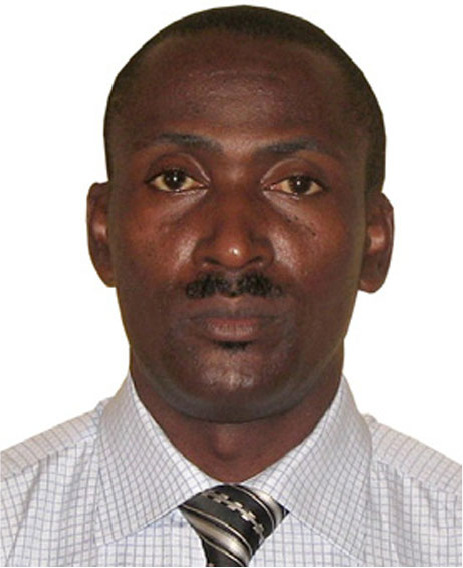



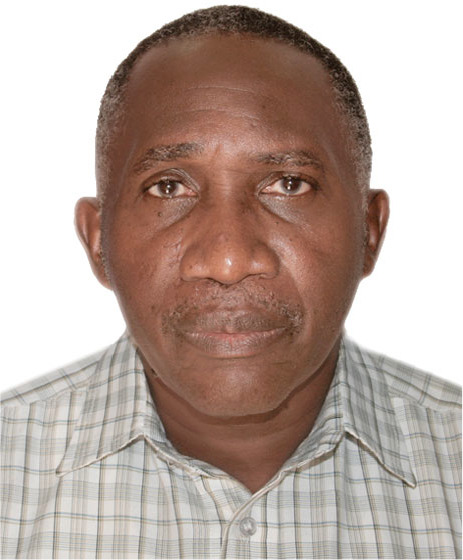



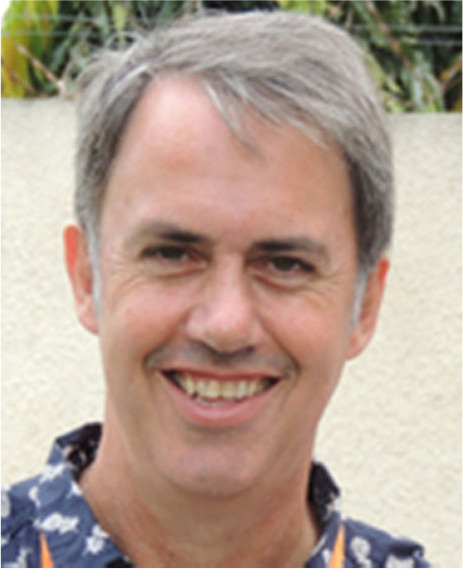



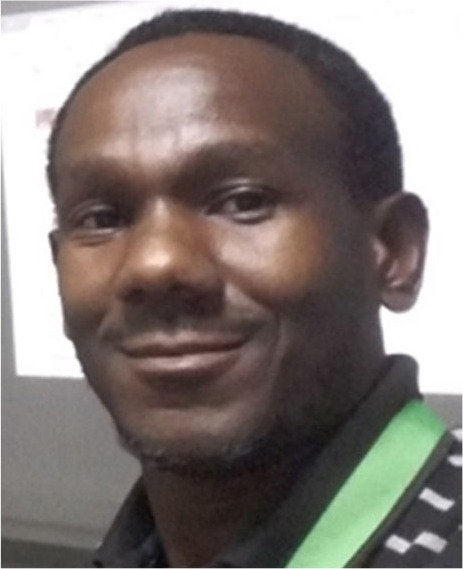



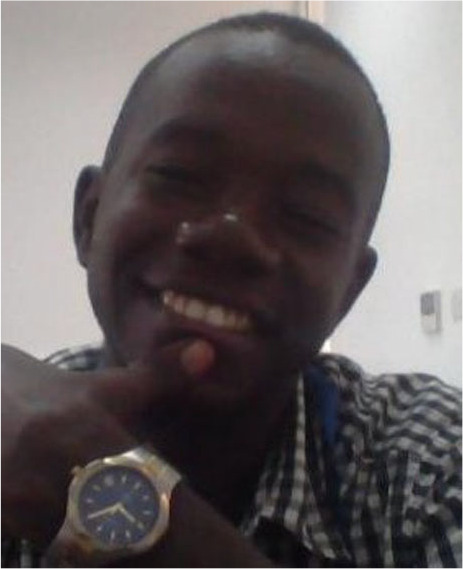



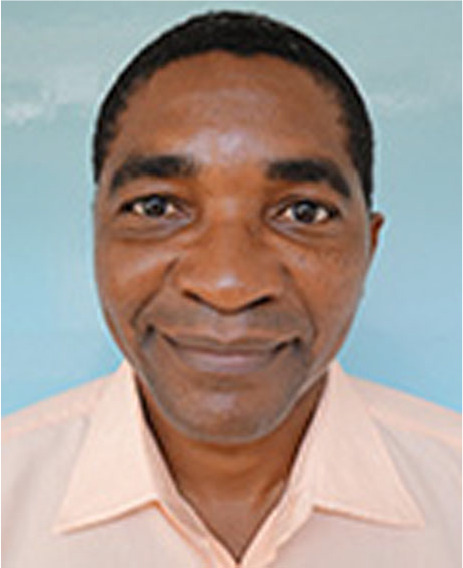



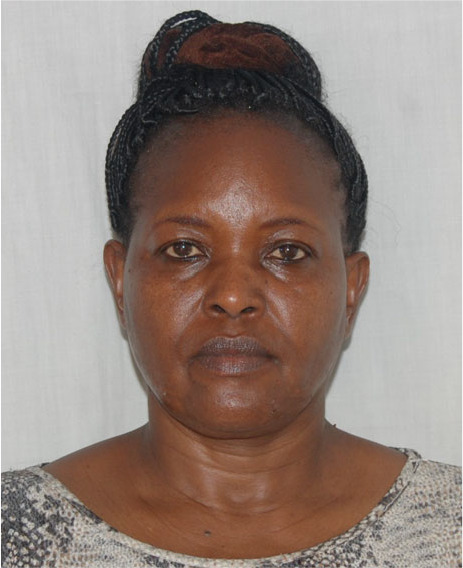



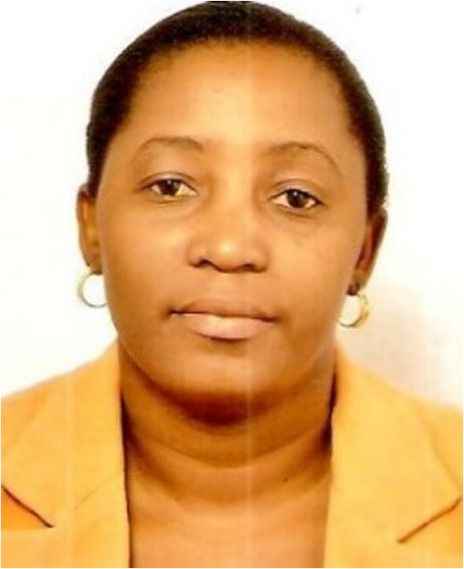



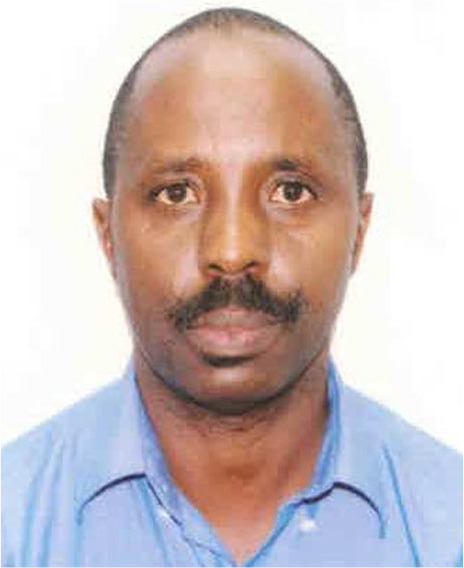



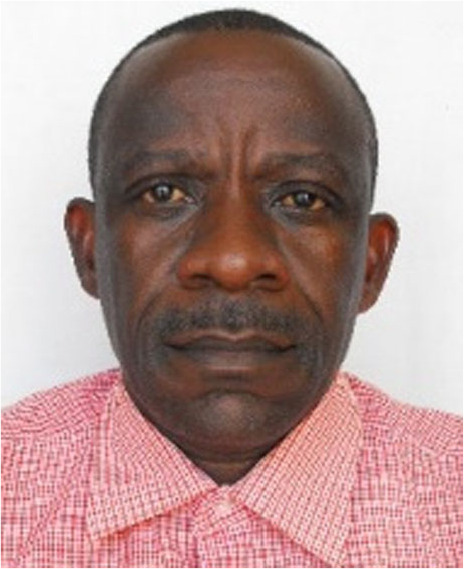



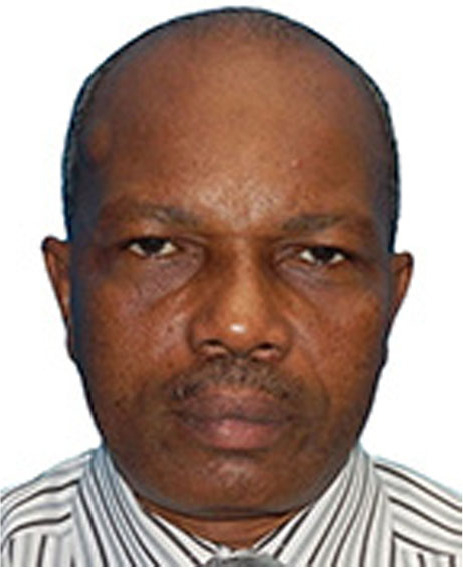



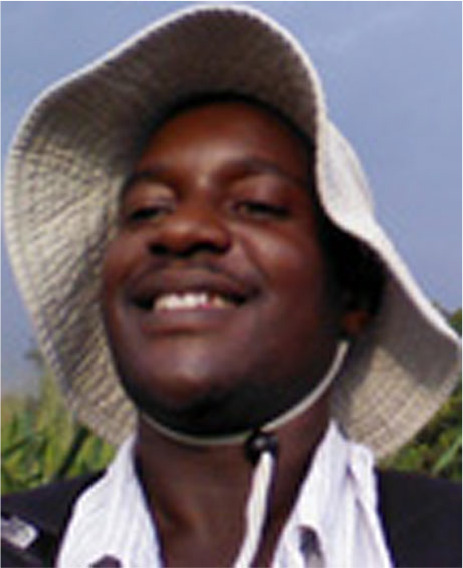



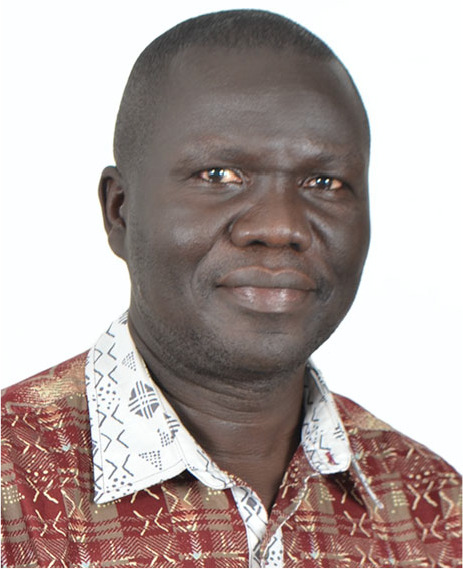



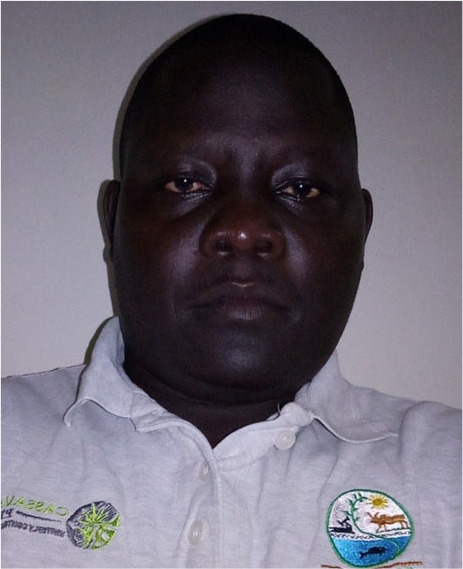



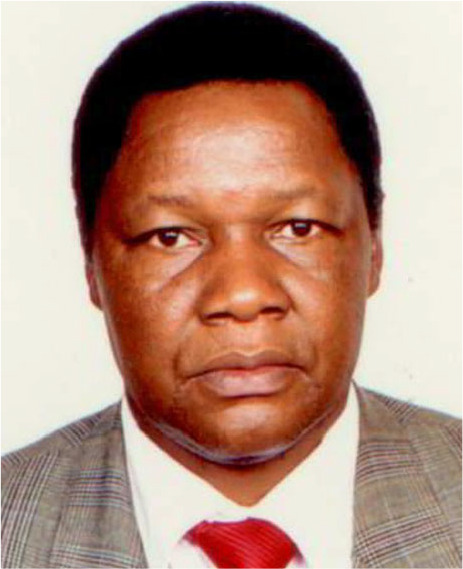



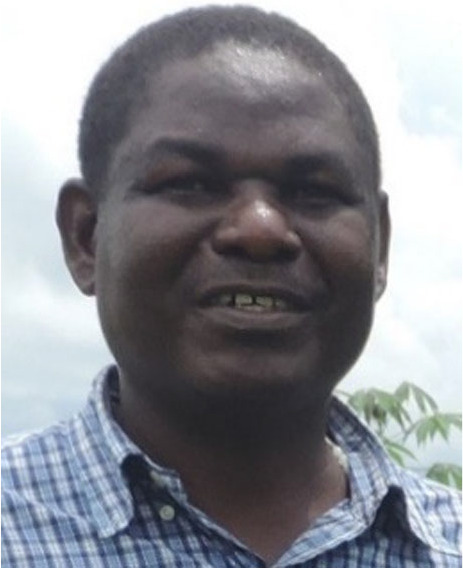



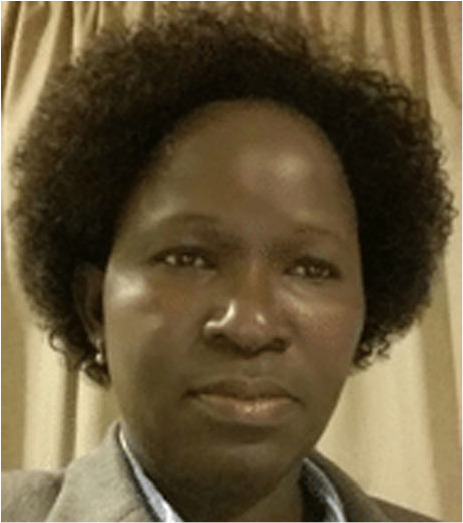



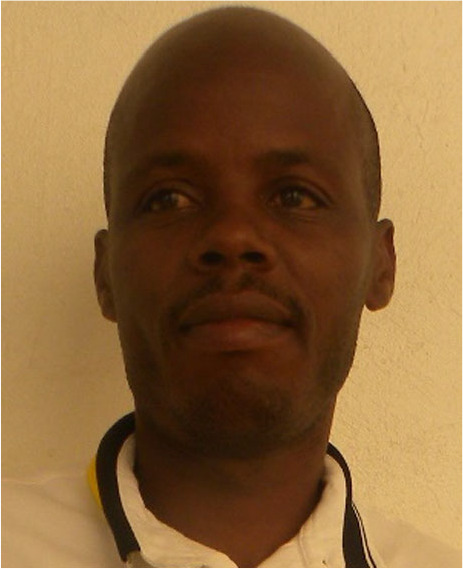



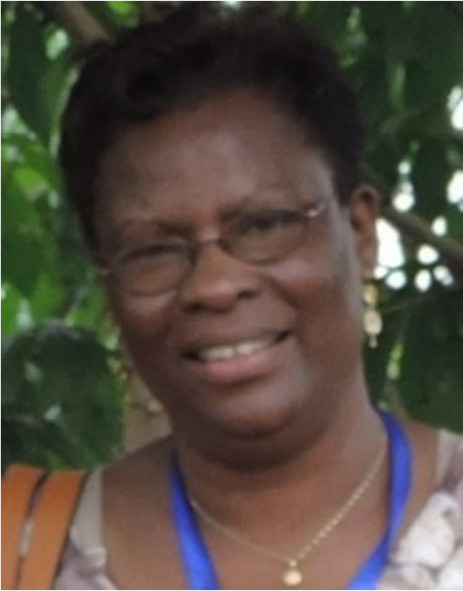



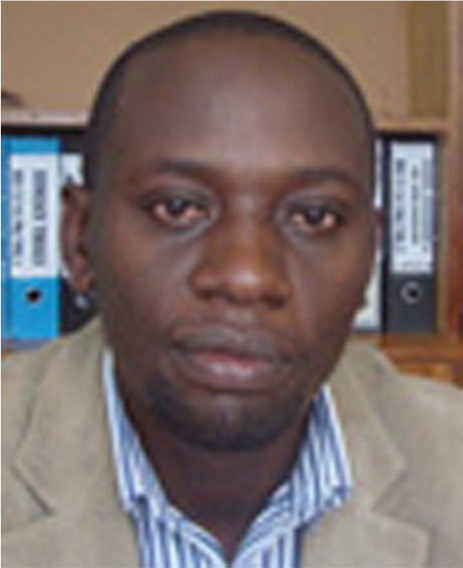



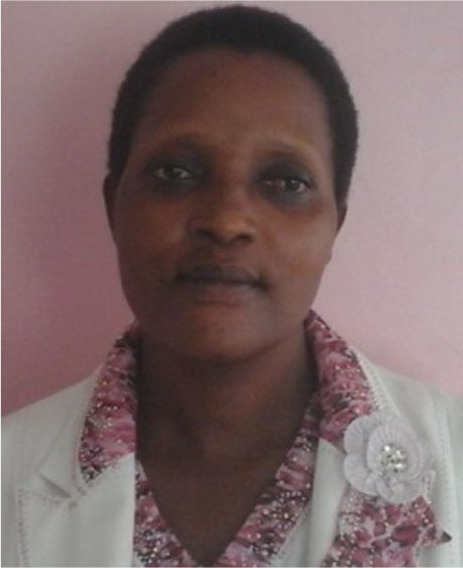



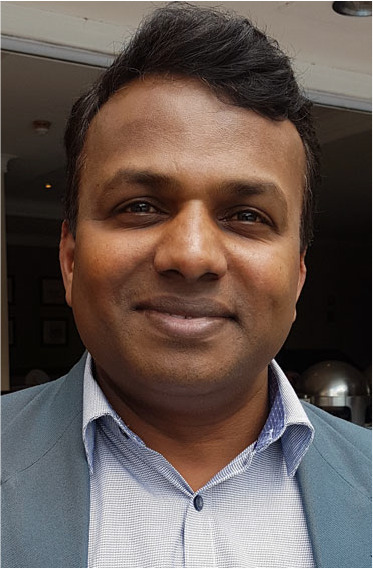



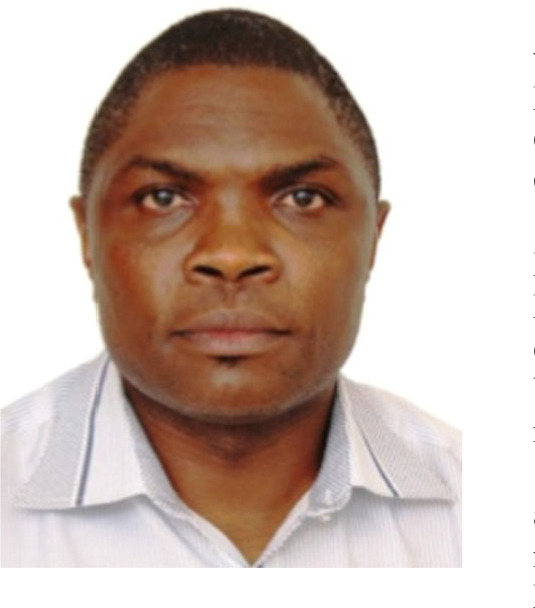



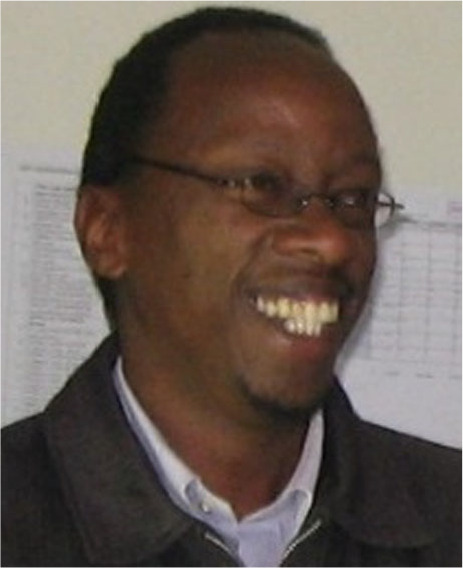



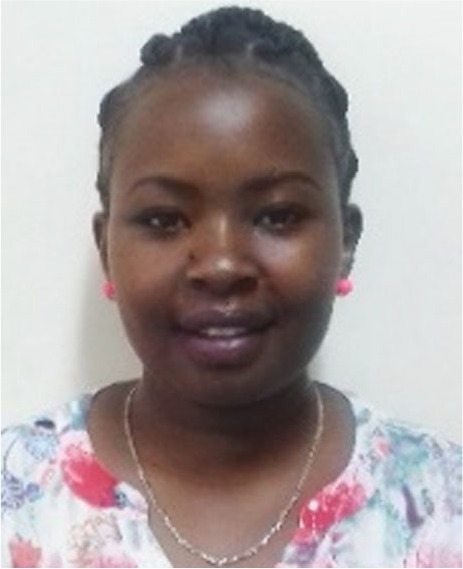



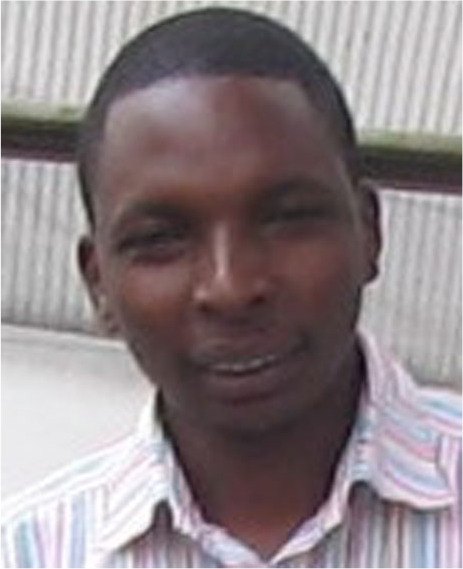



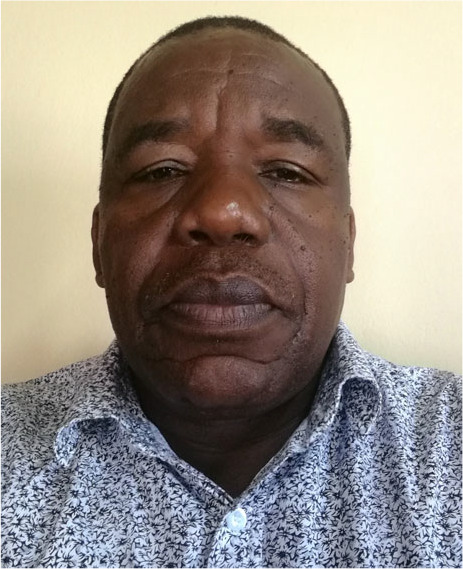



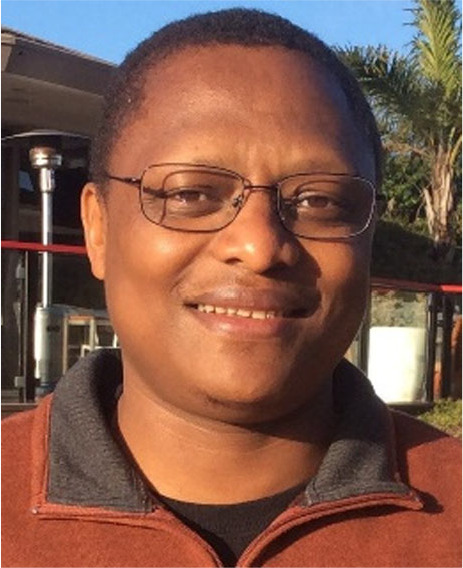



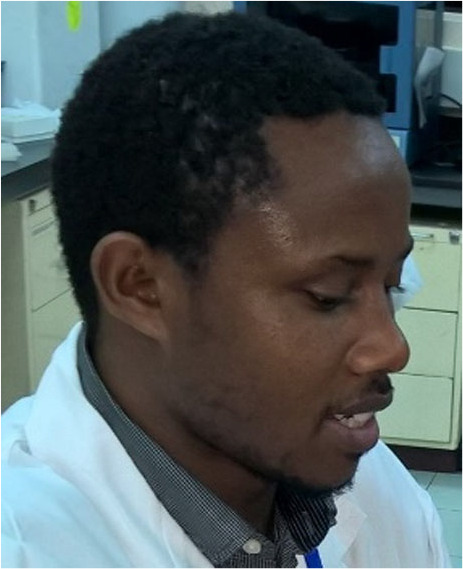


## 1 Introduction

Cassava (Manihot esculenta Crantz) is one of the most important food staples in sub-Saharan Africa (SSA), ranked as the number one root crop followed by yam and sweetpotato (FAOSTAT 2017). With over 140 MT of annual root production (FAOSTAT 2017), cassava is the major source of carbohydrates in the diet of millions of people in SSA and is grown as a famine reserve crop owing to its tolerance of harsh environmental conditions (Jarvis et al. 2012; Nassar and Ortiz 2007). Moreover, the crop has enormous potential to graduate into an important economic driver within the agriculture sector in different SSA countries where entrepreneurs have started to exploit its industrial business potential to produce high quality flour, starch, beverages and animal feeds.

However, the crop is threatened by two viral diseases: cassava brown streak disease (CBSD) and cassava mosaic disease (CMD), and these are currently the principal biotic factors affecting production in East and Southern Africa (ESA) (Alicai et al. 2007; Legg et al. 2011). While CMD is of economic importance across SSA, CBSD remains localized in ESA, although there is a high risk of the disease spreading to West Africa unless contained (Legg et al. 2011). CBSD is caused by two virus species, Cassava brown streak virus (CBSV) and Ugandan cassava brown streak virus (UCBSV) in the family Potyviridae, genus Ipomovirus (Mbanzibwa et al. 2009). Here, we refer to these collectively as cassava brown streak ipomoviruses (CBSIs). In addition to the two species groupings, several strains of each species have been reported (Mbanzibwa et al. 2009; Mohammed et al. 2012; Ogwok et al. 2015). These interact with the host differently (Mohammed et al. 2012; Kaweesi et al. 2014; Legg et al. 2015) and produce symptoms of varying severity that complicate the evaluation and selection processes in developing resistant varieties. A further important factor in the epidemiology of these viruses, causing CBSD and CMD, is the relative abundance of the whitefly vector, Bemisia tabaci (Maruthi et al. 2005). Whiteflies occur in greatly varying abundance in the regions where both diseases occur (Jeremiah et al. 2015), and this in turn results in significantly different levels of inoculum pressure for the two diseases. Consequently, it is essential to evaluate cassava germplasm that is potentially resistant to both CBSD and CMD in a wide range of agro-ecologies.

Collaborative efforts with different national cassava breeding programs have identified germplasm resistant or tolerant to CBSD/CMD. However, these have been evaluated so far under a narrow range of conditions of environment, virus species/strains, and vector abundance (Legg et al. 2011). The exchange of germplasm between countries enhances the diversity of germplasm available to partner countries. It provides breeders with fresh opportunities to evaluate and release new varieties as well as to use them as parents in efforts to breed new genotypes resistant to CBSD and CMD.

Past experiences of CMD pandemic management have underlined the significance of identifying and deploying host resistance as well as the importance of joint action among partners in the affected countries through elite germplasm exchange (Ntawuruhunga et al. 2013). Open quarantine was used effectively in efforts to manage the CMD pandemic (Mohamed 2002), but this is appropriate only under emergency conditions and where introduced germplasm is carried just a short distance over the border into the receiving country. The approach has risks of introducing other diseases/pests into new unaffected areas (Ntawuruhunga and Legg 2007). Sharing of botanical seeds is less risky, but the high level of genetic variability among individual seeds means that more time and funds are required at national level to develop promising varieties (Ntawuruhunga et al. 2013). Virus-indexed tissue culture (TC) plants are the form recognised by quarantine regulators for the exchange of elite germplasm among countries (Frison 1994; Lebot 2009; Ntawuruhunga et al. 2013).Their use also ensures that fair comparisons can be made among clones that are planted in evaluation trials without propagule-borne virus infection.

Breeding for dual resistance is currently being pursued as the most cost-effective and sustainable way to manage the devastating effects of the viral diseases in ESA. Although high resistance for CMD has been found, only limited success has been documented for CBSD. The desired goal of the breeding efforts is stable genotypes with resistance to both viral diseases. Work described in this paper has helped partner countries to access clean in-vitro stocks of a diverse set of resistant germplasm as vital precursor to coordinated regional trials aimed at identifying new resistant variety options to mitigate threats of CMD and CBSD. The work was undertaken in the early stages of the project “New Cassava varieties and Clean Seed to Combat CMD and CBSD” (5CP), and aimed at exchanging elite germplasm among the five countries most affected by CMD and CBSD for adaptability breeding.

### 1.1 Target countries

**Fig. 1:**
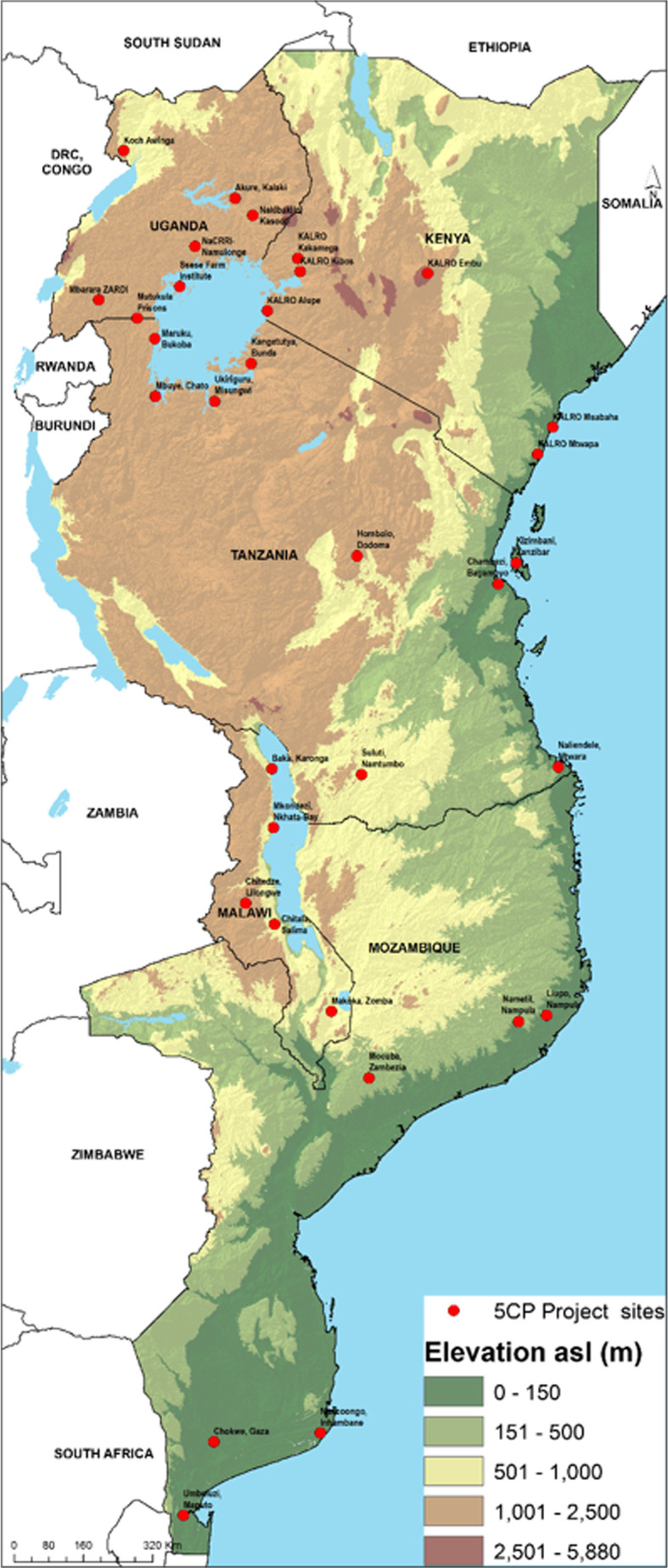
A map showing partner countries that exchanged elite cassava germplasm and sites for regional trials

Kenya, Malawi, Mozambique, Tanzania and Uganda (Fig. 1) are the countries most severely affected by both CBSD and CMD and agreed to share their five best cassava clones with respect to resistance to both diseases.

## 2 Target cassava germplasm

The targeted germplasm (Table 1) included 25 elite clones contributed by partner countries, two standard regional susceptible check clones, i.e., Kibandameno, a CMD susceptible check from Kenya, and Albert, a CBSD susceptible check from Tanzania. The elite clones were selected for exchange based on resistance to CMD and CBSD, their root yield and dry matter content. Four other clones were included as national checks as follows: Tomo (susceptible to CBSD) from Mozambique, Mbundumali (susceptible to CBSD and CMD) from Malawi, and Kiroba (susceptible to CMD) and Mkombozi (susceptible to CBSD) from Tanzania.

**Table 1:** Elite germplasm cleaned, multiplied and exchanged among five countries in eastern and southern Africa from 2012 to 2014

Variety name	Country of origin	Fresh root yield (t/ha)	DM content (%)	Reaction to diseases		Release status
									CMD resistance	CBSD resistance	
KBH2002/066	Tanzania	34.1	28.0	Moderate	Moderate	Released
Pwani	Tanzania	50.8	29.2	Moderate	Moderate	In pipeline
Mkumba	Tanzania	23.3	27	Weak	Moderate	In pipeline
KBH2006/026	Tanzania	30.0	29.0	Moderate	Moderate	Released
Kizimbani	Tanzania	28.6	28.0	Moderate	Moderate	Released
Kiroba*	Tanzania	20.0	32.0	Weak	Moderate	Released
Mkombozi*	Tanzania	>20	30	Strong	Weak	Released
Albert**	Tanzania	fair	good	Strong	Susceptible	Not released
Sangoja	Malawi	35	33	Moderate	Moderate	Released
Sauti	Malawi	30	34	Moderate	Moderate	Released
Yizaso	Malawi	35	33	Moderate	Moderate	Released
Kalawe	Malawi	28	36	Moderate	Moderate	Released
CH05/203	Malawi	33	34	Moderate	Moderate	In pipeline
Mbundumali*	Malawi	25	37	Susceptible	Susceptible	Recommended
Coliacanana	Mozambique	20.0	33.0	Weak	Moderate	Released
N’ziva	Mozambique	22.0	35.1	Weak	Moderate	Released
Okhumelela	Mozambique	20.0	32.8	Moderate	Moderate	Released
Orera	Mozambique	23.0	32.0	Weak	Moderate	Released
Eyope	Mozambique	25.0	32.0	Moderate	Moderate	Released
LML/2008/363	Kenya	69	27	Moderate	Moderate	In pipeline
F19-NL	Kenya	39.4	25	Moderate	Moderate	In pipeline
Tajirika	Kenya	61	25.7	Moderate	Moderate	Released
Shibe	Kenya	68	26	Moderate	Moderate	Released
F10–30-R2	Kenya	58	40	Moderate	Moderate	Adv. yield trial
Kibandameno^**^	Kenya	26.1	40	Susceptible	Susceptible	Not released
TZ130	Uganda	–	–	Strong	Moderate	Released
NASE14	Uganda	31.2	35.0	Strong	Moderate	Released
NASE18	Uganda	38.6	35.5	Strong	Moderate	Released
NASE1	Uganda	14.9	32.5	Strong	Moderate	Released
NASE3	Uganda	<10	30.0	Moderate	Moderate	Released

* National susceptible checks returned only to countries of origin; ** Standard susceptible checks distributed with the elite clones

### 2.1 Partner institutions

IITA led the initiative through its Eastern Africa Hub in Dar es Salaam Tanzania. The five partner countries were represented as follows: Uganda by the National Agricultural Research Organization (NARO), Kenya by the Kenya Agricultural and Livestock Research Organisation (KALRO), Tanzania by the Department of Research and Development, (DRD), Mozambique by Instituto de Investigação Agrária de Moçambique (Agricultural Research Institute of Mozambique -IIAM), and Malawi by the Department for Agricultural Research Services (DARS). Other partner institutions were the Natural Resources Research Institute (NRI) in the United Kingdom, Kenya Plant Health Inspectorate Services (KEPHIS) and GTIL in Kenya.

### 2.2 Main steps for the exchange and management of elite cassava germplasm

**Fig. 2:**
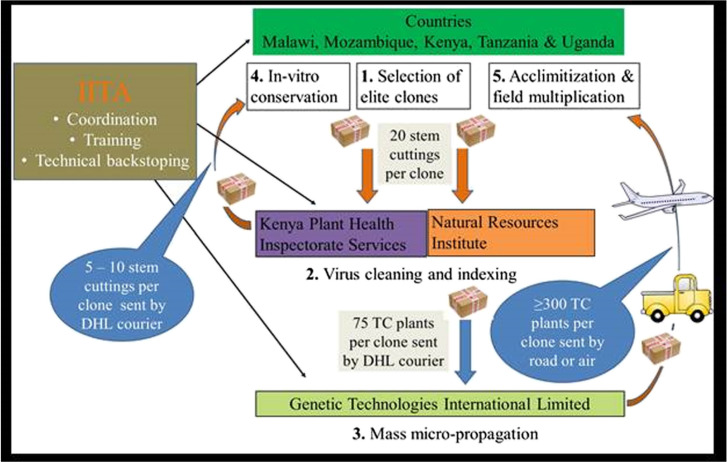
Schematic flow of the cleaning, multiplication and exchange process of the cassava elite germplasm

**Fig. 3:**
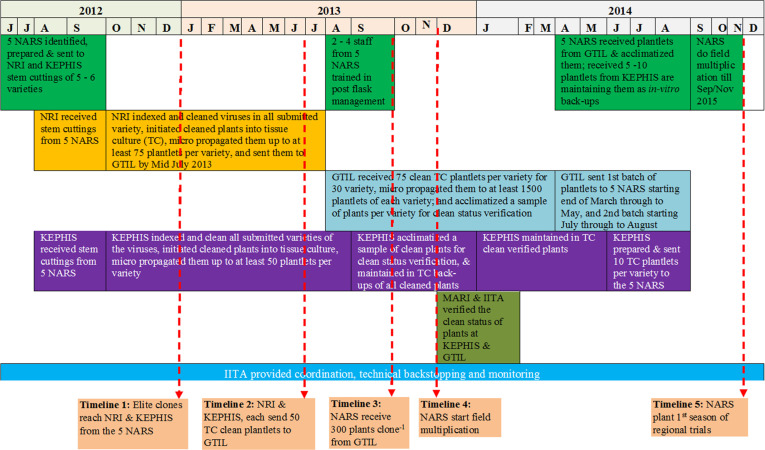
Timelines for the process of cleaning, multiplication and exchange of the cassava elite germplasm

Four main steps (Fig. 2) were undertaken in the process of exchanging and managing the elite germplasm in the target countries. Figure 3 illustrates the timelines.

### 2.3 Obtaining elite cassava clones from partner countries

Between August and December 2012 each country freely agreed to submit to NRI and KEPHIS ca 25 stem cuttings obtained from asymptomatic plants of each of the best five clones (Table 1) for virus cleaning. The asymptomatic plants were used mainly to ease the task and cost of the cleaning process. To minimize chances of within-clone variation (mericlones), stem cuttings of a given clone were collected from one plant stand by breeders who had wide knowledge of the clones’ agronomic performance as well as their reaction to the diseases. The two laboratories served as a backup to each other in the role of virus cleaning and indexing. Apart fromshort delays in the delivery of the elite clones by some countries, no major challenge was experienced during this step of the process.

### 2.4 Virus cleaning and indexing

At each laboratory, the stem cuttings were sprouted and grown in pots under quarantine for about 3 months while being observed for virus disease symptoms. At NRI, the asymptomatic plants were separated from the symptomatic (data not presented), and leaf samples from the former were subjected to virus diagnostics using PCR procedures (Aloyce et al. 2013) for cassava mosaic begomoviruses (CMBs) and real-time reverse transcription polymerase chain reaction (real-time RT PCR) procedures (Adams et al. 2013; Tomlinson et al. 2013) for CBSIs. The virus negative plants of each clone were initiated into TC and micro-propagated to raise over 50 plantlets. Meristems were used to initiate TC plants for those clones with only virus positive plants. These were incubated under thermotherapy conditions and left to grow for 8–10 weeks before being reintroduced to the quarantine glasshouse to confirm the absence of the viruses using the diagnostic methods indicated above. At KEPHIS, meristem tips of asymptomatic plants were initiated into TC and later diagnosed for viruses. As at NRI, the virus negative plants were micro-propagated to raise over 50 plantlets. Virus positive plants were incubated in the thermotherapy chamber at 38 °C for 21 days before meristems were chosen for TC initiation. Upon establishment, the plants were tested again for viruses, and the cycle was repeated for the positive plants until negative plants were obtained and micropropagated to produce over 50 plantlets.

### 2.5 Preparation and dispatch of elite clones

This step involved five activities: micro-propagation, verification of genotype purity and virus-free status, capacity building, mock shipment and shipment of TC plants to partner countries.

**Photo 1:**
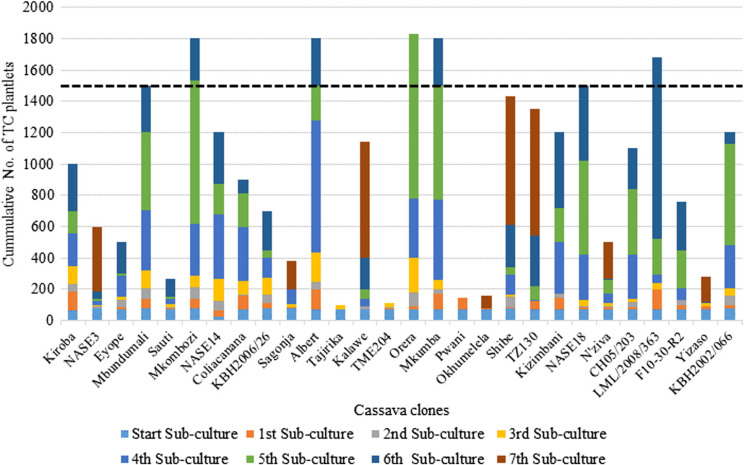
**a** micro-propagation at Genetic Technologies International Limited; **b** acclimitization work at Kibaha; **c** field multiplication of elite germplasmat Kandiyani,Malawi; and **d** partners of breeders and virologists in a cassava field in Malawi

**Fig. 4:**
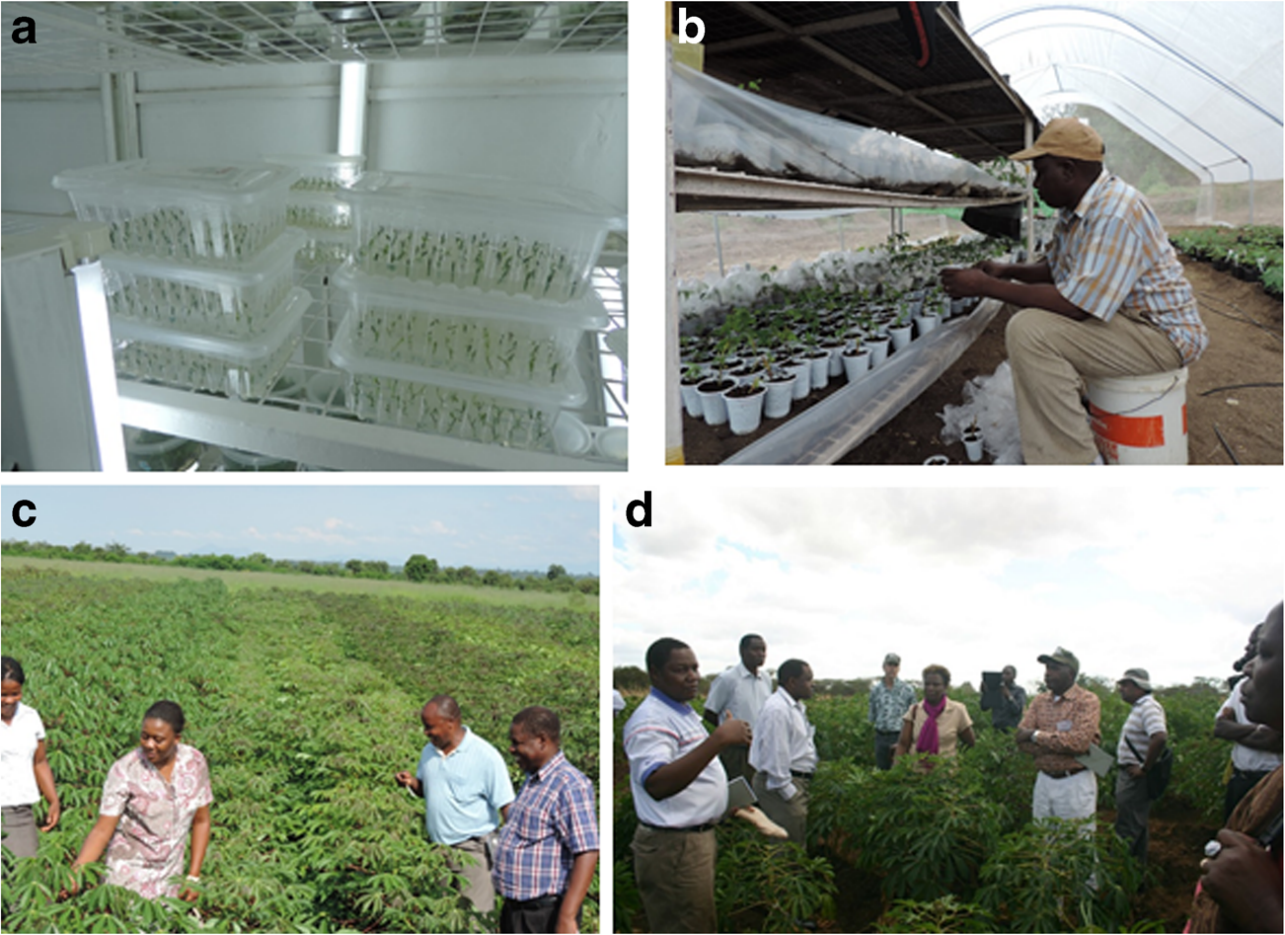
Variation in in-vitro micro-propagation rates of different cassava clones at GTIL tissue culture lab, Nairobi. Sub-culturing was done after every 3 weeks of growth in-vitro
b) **Verification of genotype purity and virus status of the clones**: It was considered critical for independent parties to ascertain the genotype purity and virus-free status of the clones before shipment to partner countries. About ten sample plants were acclimatized at GTIL (for materials cleaned by NRI) and at KEPHIS from which later samples of different plant parts (leaves, stem and meristem) were taken and tested for CBSIs and CMBs by Mikocheni Agricultural Research Institute (MARI) in collaboration with IITA. For verification of genotype purity more samples were collected by Biosciences eastern and central Africa (BecA) for fingerprinting. The results of the virus-free status verification are shown (Table 2). Almost all the clones were confirmed to be free of both CBSIs and CMBs. Positive virus tests were recorded for three clones at KEPHIS. Plants associated with virus positive samples were discarded, and virus negative plants were retained. Fingerprinting results (not shown) also confirmed true-to-type genotypes between clone plants handled at the two laboratories.

a) **Micro-propagation**: This was done by GTIL a private TC laboratory in Nairobi, Kenya, to increase the number of clean plantlets per clone available to partner countries. From NRI, approximately 75 TC plants per clone were sent to GTIL and micro-propagated (Photo 1a) to produce more than 1500 plantlets per clone, which were sufficient to provide at least 300 plantlets per clone per country. Figure 4 shows the rates of progress of micropropagation at GTIL. While most clones responded positively to the protocols and yielded the required quantities by sub-culturing cycle 5 or 6, others such as Pwani, NASE 1, Tajirika, and Okhumelela were recalcitrant and required media improvement. The recalcitrant clones caused delays and held back the timelines for delivery of the plants to partner countries (Fig. 3)

**Table 2:** Virus clean status verification results of TC-derived plants of different cassava clones targeted for regional exchange among five countries in eastern and southern Africa

Clone	Status of NRI sample plants				Status of KEPHIS sample plants			
	No. of samples	CBSV*	UCBSV*	CMBs	No. of samples	CBSV*	UCBSV*	CMBs
CHO5/203	6	─	─	─	4	─	─	─
NASE18	4	─	─	─	7	─	─	─
Tajirika	12	─	─	─	3	─	─	─
Albert	16	─	─	─	nd	nd	nd	nd
TME204	5	─	─	─	3	─	─	─
Kalawe	24	─	─	─	24	─	─	─
Orera	9	─	─	─	11	─	─	─
Coliacanana	5	─	─	─	18	─	─	─
NASE14	3	─	─	─	3	─	─	─
KBH2006/026	3	─	─	─	9	─	─	─
Sagonja	2	─	─	─	2	─	─	─
Okhumelela	9	─	─	─	7	─	─	─
SHIBE	17	─	─	─	14	─	─	─
TZ130	46	─	─	─	2	─	─	─
Pwani	9	─	─	─	3	+++	─	─
Mkumba	3	─	─	─	17	─	─	─
Yizaso	6	─	─	─	39	─	+	─
LML/2008/363	4	─	─	─	3	─	─	─
F10–30-R2	6	─	─	─	8	─	─	─
Kiroba	3	─	─	─	nd	nd	nd	nd
Mbundumali	21	─	─	─	18	─	─	─
N’ziva	8	─	─	─	12	─	─	─
Sauti	18	─	─	─	2	─	─	─
Eyope	11	─	─	─	16	─	─	─
NASE3	10	─	─	─	1	─	─	─
Kizimbani	1	─	─	─	3	─	─	─
F19-NL	nd	nd	nd	nd	4	─	─	─
KBH2002/066	nd	nd	nd	nd	11	─	─	─
72 TME14	nd	nd	nd	nd	3	─	++	─
Total	261				248			

* ─ = negative result, + = one sample positive result, ++ = two sample positive results; +++ = three sample positive results; nd = not determined
c) **Capacity building (Capacity preparedness)**: IITA, in collaboration with Uganda’s cassava research program (also a regional Centre of Excellence for Cassava Research) at the National Crops Resources Research Institute (NaCRRI), conducted a 2-week practical training on post-flask management. Each country (except Tanzania, which sent four technicians) was represented by two senior technicians who formed the core team to receive and manage the TC plantlets in their countries. Also, two staff from KEPHIS and GTIL each participated in the training. The trainees were equipped with both theoretical and practical knowledge and skills for preparing and handling TC plants for acclimatization and multiplication purposes. The key infrastructure required was the TC laboratory for in-vitro conservation of clean stocks and insect-proof screen houses for acclimatization work. No improvements were made to the TC laboratories, but screen houses in Malawi and Mozambique were repaired and new ones constructed in Kenya (for KEPHIS) and Tanzania. All these steps were undertaken to ensure that countries were ready and prepared to receive material.
d) **Mock shipment**: GTIL undertook a mock shipment prior to the actual shipment as a way of testing the readiness of the partners to receive the materials, as well as of identifying possible challenges during shipment. Two cuboid plastic containers (Photo 1a) each with a maximumof 150 plantlets of one clone were sent by courier to Uganda, Tanzania, and Malawi. Permits for both export and import accompanied the shipment as required by regulations. Two challenges were experienced during mock shipment and recommendations were made to GTIL for the actual shipment. Firstly, 150 plantlets per container were overcrowded with intertwined roots that resulted in both root and plant damage during removal from the media. A total of 75 TC plants per container was recommended for the actual shipment. Secondly, all countries reported plant damage resulting from the semi-solid media becoming loose and mixed with plants during shipment. GTIL was asked to use a more solidified medium to ensure that plants would be kept in position during shipment and to improve the placement of the containers in the big boxes. The mock shipment experience also led to GTIL being advised to ensure that consignments were projected to arrive in target countries early in the week for easy clearance and to avoid shipments being held up over weekends. Also, improvements were made to the system used to alert recipient countries about the expected timetable for dispatch of the TC shipments from GTIL and incountry arrival. This enabled national partners to make adequate preparations for receiving the material through liaising with authorities involved in the clearing process.
e) **Shipment of TC plants**: This is the activity that marked the exchange of the germplasm, between partner countries. The first and major shipment was undertaken by GTIL. Using cylindrical or cuboid plastic transparent containers, GTIL planted in the medium a maximum of 75 plantlets per container, labelled them, allowed two weeks for roots and shoots to develop, sealed, packaged, and sent the plantlets to the partner countries. The containers were placed in bigger boxes and surrounded with shockabsorbing materials. The boxes were also marked fragile and an ‘up’ arrow sign was used to ensure proper handling during transit. The plants were transported by road to Uganda and Tanzania or as cargo with international airlines to Malawi and Mozambique. Kenya, unlike other countries, received hardened plants from GTIL. To facilitate quarantine authority clearance at entry points, all the shipments were accompanied by copies of import permits that were obtained from plant protection offices in respective countries and phytosanitary certificates from KEPHIS. At the point of entry, the consignments were cleared by officials from the national plant protection organizations (NPPOs) of the respective countries and received by the national cassava research teams.

The second shipment was made by KEPHIS, where 5 to 10 TC plantlets per clone in single glass test-tubes were shipped to partner countries. The purpose of these plantlets was to be conserved as back-ups for immediate post-flask management needs and also for future clean stock needs.

### 2.6 Management of the exchanged elite clones in target countries

a) **Post-flask management**: Upon arrival in each country, the TC plantlets were checked for contamination and physical damage, and registered. The plantlets were kept for two to seven days in TC laboratory growth rooms or under normal room temperature conditions to recover from transit stress. They were then carefully separated from the medium, introduced into individual small cups filled with vermiculite or forest soil/sand mixture, and placed in nutrient-enriched water baths. Each potted plant was covered with a transparent polythene bag to create micro-humid conditions. In Tanzania, the potted plants covered with polythene bags were additionally placed in a bigger humidity chamber. After one week, the polythene bags were cut open at one end and fully opened after two weeks (Photo 1b). The tender plants were sprayed with fungicide and insecticide and irrigated with nutrient-enriched water. After a month, the plants were transplanted into bigger polythene bag containers (also used in potting tree and flower seedlings) filled with sterilized soil. By the end of the second month the plants were ready for transplanting into the field for multiplication. Different rates of survival were registered by the different countries (Table 3). Uganda (79.4%) and Mozambique (80.9%) registered the highest survival rates with fewer losses than Malawi, Kenya. and Tanzania. There had been heavy losses in the first batch of delivered plants but survival improved with the second batch.

**Table 3:** Achieved survival rates of TC plantlets during post-flask management in different countries during 2014

Country	No. clones received	Total No. plants sent by GTIL	No. of plants after post-flask management	Average survival rate (%)
Uganda	27	7650	6074	79.4
Kenya	23	13,050	7099	54.4
Malawi	27	10,775	6433	59.7
Mozambique	27	8075	6533	80.9
Tanzania	28	11,450	6091	53.2
b) **Field multiplication**: The fields used for multiplication were located in areas with very low CBSD and CMD pressure. Hardened plantlets were established in one multiplication field (Photo 1c) in all countries except Tanzania where two fields were used in different parts of the country. The management practices varied with countries. For example, irrigation was done during periods of drought in Kenya, Tanzania, and Mozambique. In Malawi, the severe cold period between June and October 2014 delayed further establishment of the multiplication field to avoid plant losses to frost. In Uganda, field multiplication was rain fed and no fertilizer application was made. To minimize any virus infection, the multiplication fields were isolated by being at least 200 m from any old cassava crop and continuously rogued.
c) **Macro-propagation**: To mitigate the challenge of plant losses during post-flask management and ensure sufficient numbers of hardened plantlets for field multiplication, two- to three-node cuttings were taken from the few surviving plants and planted for further multiplication. In the screen houses, these were hardened plants between two and three months old and ready for transfer to the field. In the multiplication fields, these were plants at four to six months after establishment. In both cases, the cut plants sprouted with multiple shoots in addition to three or more plantlets generated from stem cuttings. Through this technique, the countries that suffered early plant deaths during acclimitization were able to increase the number of plants of the affected clones. The technique helped to save time and reduce costs of acquiring and acclimatizing new sets of TC plants from GTIL by the affected countries. It also ensured that there were sufficient stem cuttings of at least 20 elite clones for regional trials to be established by the end of 2015 in all countries. This technique can also be helpful in multiplication of hardened virus-indexed cassava plants under insect-proof screen houses for pre-basic seed production under cassava seed systems (IITA 2014).
d) **Regional multi-locational trials**: The immediate purpose of exchanging the elite germplasm between countries as discussed in this paper was to test and validate the clones’ adaptability and tolerance to CBSD and CMD across five partner countries all severely affected by CBSD and CMD. Using stem cuttings obtained from the multiplication field discussed above, field trials were established in a total of 33 sites across the partner countries (Fig. 1). The sites were characterized by varying levels of CBSD and CMD pressure, climatic and soil conditions. With the exception of Kenya, the countries successfully established trials with all the 25 elite clones and standard susceptible checks (Albert, a susceptible check for CBSD and Kibandamendo, a susceptible check for CMD). In Kenya, only 23 clones were established in the trials and four clones (N’ziva, Okhumelela, Shibe, and Albert) were omitted owing to plant losses during the post-flask management stage. Comprehensive data were collected for agronomic traits, viral disease incidence and severity at different stages of crop growth (including harvesting), whitefly abundance, and yield components. The data will be analysed in the near future and are expected to help in understanding the genotype by environment interactions for CBSD/CMD resistance. Also, the data will be helpful in identifying superior clone/varieties and environments for the evaluation of resistance to CBSD/CMD.
e) **In-vitro conservation**: The clean plants received from KEPHIS in all countries were meant to be conserved as in-vitro back-ups to serve both immediate needs to replace plantlets of any clone lost during the acclimatization process and long-term needs for clean stocks for pre-basic seed production systems of identified varieties in each country. Apart from Uganda and Kenya, the countries reported the loss of all or the majority of the clones (Table 4). Erratic power supplies faced by most NARS in TC facilities and the lack of TC reagent supplies were reported as the main causes of plant losses for the clones. Additional investments in tissue culture facilities in the countries where these losses occurred will be required in future in order to allow for long-term TC-based germplasm conservation.

**Table 4:** Survival rates (%) of in-vitro backup plantlets at different national tissue culture laboratories

Country	No. of clones received from KEPHIS	Tissue culture lab location	No. of clones in conservation at 31.12.2016	% clone survival	Remarks
Malawi	27	DARS Vumbwe	0	0	Lost all clones due to power cuts and lack of TC reagents for conservation
Mozambique	26	IIAM Maputo	10	38.5	Lost some clones due to power cuts
Kenya	31	KEPHIS	31	100	TC conservation part of their mandate and have personnel
Uganda	27	NaCRRI	27	100	Have a functional TC lab with personnel
Tanzania	24	MARI/CBS	14	25.6	MARI lost all clones due to power cuts. The surviving clones are with CBS

### 2.7 Key achievements

There were three major acheivements under the intiative reported here. Firstly, up to 30 clones (25 elite, two standard checks and four national checks) were successfully virus-cleaned and indexed, and 27 cloneswere exchanged among target countries. The other three were returned to their respective mother countries as national checks. The particularly unique aspect of this initiative was the sourcing and pooling of germplasm from five countries, the cleaning, and returning of the expanded pooled set. Additionally, the germplasm presented a unique opportunity to identify varieties with high levels of resistance to both CBSD and CMD under the diverse range of virus/virus vector/ environmental conditions in these five countries of ESA. In addition to offering potential for use as parents to generate superior progeny with a background of high resistance, the clean stocks represented a great asset for initiating extension programs for the multiplication and dissemination of high quality pre-basic ‘seeds’. Hitherto, most of the target countries have had no access to field-based stocks of high quality virus-tested planting material.

The second achievement is the strong partnership built between breeders and virologists in national and international institutions (Fig. 2; Photo 1d) that has ensured the successful exchange of elite germplasm. It represents a joint action to reverse the devastating effects of the two deadly viral diseases. The partnership also allowed cross-learning between partners at all levels of the process. It has presented a unique opportunity for breeders and virologists to work together to combat these diseases through elite resistant germplasm and clean seeds. Legg et al. (2014) previously highlighed the importance of concerted multi-partner efforts to tackle the twin threats of CBSD and CMD to Africa’s cassava production. The current initiative represented a significant achievement along the road map proposed for the ‘War on Cassava Viruses’ that was described in this publication. However, a key question is whether the partnership will continue after the project. Partner institutions will need to make concerted efforts to ensure that these linkages are sustained, and furthermore, that the lessons learned from the approach are shared with others who are faced with similar challenges affecting vegetatively propagated crops.

The third achievement was the successful implementation of the trials across the countries. Following analysis, the collected data are anticipated to elucidate the magnitude of genotype by environment interactions for CBSD/CMD resistance, superior clones, and environments. Although these trials are still in progress, preliminary results are already helping to guide stakeholders on which varieties are likely to be most appropriate in which agro-ecologies and CBSD/CMD disease pressure conditions within the eastern and southern African region. Moreover, these preliminary data and the germplasm exchange approach described here are currently being used to inform the implementation of new CBSD mitigation programs in Burundi, Rwanda and the eastern Democratic Republic of Congo.

### 2.8 Learning experiences

- Cleaning from virus infection was relatively easy for about 85% of the cassava clones. These were cleaned in the ‘first cycle’ of the virus indexing program and were thereafter ready for micro-propagation. This success was possibly due to the selection and use of stem cuttings obtained from asymptomatic plants by the breeders. The remaining 15% of the clones took about three cycles, i.e., three treatments of chemotherapy, thermotherapy, and tissue culturing, before becoming clear of viruses. These clones were Tomo, F19-NL, NASE1 and Kibandameno; all were symptomatic after sprouting at NRI/KEPHIS. Only Tomo, a check clone from Mozambique, could not be cleaned at KEPHIS despite several repeated cycles. Starting a virus clean-up process such as this with visually clean plants directly influences how quickly the target clones can be cleaned of viruses. However, specificity in the response of cassava genotypes to the cleaning and indexing protocols has been reported (Sesay et al. 2016). Future initiatives attempting similar work should recognize that a small proportion of plant genotypes are likely to be recalcitrant to virus eradication.
- Responses of clones to micro-propagation protocols were varied at GTIL (Fig. 3). Although the majority responded positively to the protocols and yielded the required quantities by sub-culturing cycle 5 or 6, others such as Pwani, NASE1, Tajirika, and Okhumelela were recalcitrant and optimization of the medium was needed. The recalcitrant clones also caused delays and held up the timelines for delivery of the plants to countries (Fig. 2). TC recalcitrant behaviour among clones has previously been reported in cassava (Acedo and Corazon 2008). Further investigation is needed to establish the optimal protocol for micropropagation of most of the genotypes.
- Extreme temperatures adversely affected the plants while being acclimatized. For example, extremely cold night temperatures in Nairobi either slowed growth or killed the plants in Kenya. On the other hand, hot screen house conditions scorched some of the plants to death at Kibaha and Maruku in Tanzania. These experiences suggest that temperature conditions are important in selecting a station where acclimatization of cassava plants should be conducted. Specifically, the choice is recommended of shaded cool or fresh environments with no extreme temperatures (FAO 2010). In Tanzania, the extremely hot conditions were overcome by introducing a shade net inside the screen house but this could also be overlaid on top of the white insect-proof net.
- Bulk packaging of the TC plantlets (75 per container) at GTIL was cost-effective but presented several challenges. Firstly, the plantlets were exposed to contamination during shipment owing to ineffective sealing of large containers. Secondly, the plantlets were vulnerable to damage during removal for hardening. The roots of the plantlets grow and intertwine into a network, making it difficult to separate them without root or plantlet damage. Thirdly, for some clones (e.g., Tajirika) the plantlets in the middle of the container tended to be slow in developing roots resulting in plantlets with no roots or underdeveloped roots that had a high chance of early death during acclimatization. Single test-tube TC plant packaging is the commonly used method and is associated with limited contamination and damage during removal. However, testtubes can be expensive and bulky.
- It was critical to have skilled and committed personnel to monitor the acclimatizing plants carefully and constantly, closely observing the progress of each plant. Therefore, training and supervision of personnel are vital. Best results were achieved in countries where the monitoring tasks were assigned to one committed technician.
- We experienced variation across countries in capacities for in-vitro conservation of elite germplasm. During the course of the initiative, most partner countries except Uganda and Kenya (where plants were kept at KEPHIS) lost more than half of the in-vitro back-up plants from infrastructural difficulties such as the lack of reliable and stable electricity as well as of reagent supplies. Future initiatives should therefore pay particular attention to minimising the likelihood of infrastructural constraints having a negative impact on the work.
- The coordination role played by IITA (Fig. 2) contributed greatly to the success of the entire exercise. The project leadership ensured effective communication, and the preparation of the different partners. Prior alerts of shipment schedules and departures to recipient countries were helpful in clearing the materials through plant health inspection offices at entry points and avoiding possible delays, as well as for country teams in making all the needed preparations.

## 3 Conclusion

This initiative is the first of its kind in successfully facilitating the exchange of a large number of clean virusindexed elite germplasm clones to combat CBSD and CMD. However, the success achieved was the result of extensive preparation of infrastructure and trained personnel to manage the different sub-processes in the framework of an effective partnership. This could have been achieved only within the context of a large project, which in this case was supported through a grant from the Bill and Melinda Gates Foundation. Even where such project support may not be available in future years, the approach has provided a valuable model for other national, regional, and international stakeholders in African agricultural development that may have an interest in supporting similar initiatives in future. Although the programme achieved its goals, some important challenges were identified. In particular, it was noted that a small proportion of cassava genotypes were recalcitrant either to virus cleaning or micropropagation, and these slowed down the overall process. However, more than 80% of the genotypes used responded well to all stages of the process and by the end of the exercise, one of the most extensive cassava germplasm evaluation trials ever undertaken was underway at more than 30 locations in the five target countries of ESA. The trial will allow the region’s cassava researchers to assess the performance of this elite set of varieties under a diverse set of agro-ecological conditions, and to characterize varietal responses to the viruses causing CBSD and CMD under each of these conditions. This in itself will be a unique undertaking, and should provide a lasting legacy for cassava development and efforts to combat these diseases in one of the most important cassava-growing regions of Africa. In view of the importance of cassava to Africa’s food security given the threats posed by future climate change (Jarvis et al. 2012), this represents a very significant contribution.

## Abbreviations

BecA: Biosciences Eastern and Central Africa
CBSD: Cassava Brown Streak Disease
CBSV: Cassava Brown Streak Virus
CMD: Cassava Mosaic Disease
CMB: Cassava Mosaic Begomovirus
DARS: Department of Agricultural Research Services
DRD: Department of Research and Development
ESA: Eastern and Southern Africa
GTIL: Genetic Technologies International, Limited
IIAM: Instituto de Investigacao Agraria de Mocambique
KALRO: Kenya Agricultural and Livestock Research Organisation
KEPHIS: Kenya Plant Health Inspectorate Services
MARI: Mikocheni Agricultural Research Institute
NaCRRI: National Crop Resources Research Institute
NARO: National Agricultural Research Organization, Uganda
NRI: Natural Resources Institute
TC: Tissue Culture
PCR: Polymerase Chain Reaction
NARS: National Agricultural Research Systems
CBS: Crop Bioscience Solutions
